# Multifunctional graphene supports for electron cryomicroscopy

**DOI:** 10.1073/pnas.1904766116

**Published:** 2019-05-24

**Authors:** Katerina Naydenova, Mathew J. Peet, Christopher J. Russo

**Affiliations:** ^a^Medical Research Council Laboratory of Molecular Biology, Cambridge, CB2 0QH, United Kingdom

**Keywords:** cryoEM, structure determination, graphene functionalization, low-energy plasma

## Abstract

With recent technological advances, the atomic resolution structure of any purified biomolecular complex can, in principle, be determined by single-particle electron cryomicroscopy (cryoEM). In practice, the primary barrier to structure determination is the preparation of a frozen specimen suitable for high-resolution imaging. To address this, we present a multifunctional specimen support for cryoEM, comprising large-crystal monolayer graphene suspended across the surface of an ultrastable gold specimen support. Using a low-energy plasma surface modification system, we tune the surface of this support to the specimen by patterning a range of covalent functionalizations across the graphene layer on a single grid. This support design reduces specimen movement during imaging, improves image quality, and allows high-resolution structure determination with a minimum of material and data.

## Significance

Single-particle electron cryomicroscopy (cryoEM) has now proved to be the method of choice for determining the structure of biological macromolecules and complexes. Yet success in determining a structure by cryoEM depends on being able to prepare a frozen specimen on a small metal support called a grid. This process is poorly controlled at present because of molecule–surface interactions. Here we used a modified form of graphene, in conjunction with a stable grid made of gold, to control these surface effects. Functionalized graphene-on-gold grids improve the reliability of specimen preparation and enhance image quality. This technology has the potential to take specimen preparation for cryoEM from a trial and error art to a controlled and reproducible process.

**Fig. 1.**
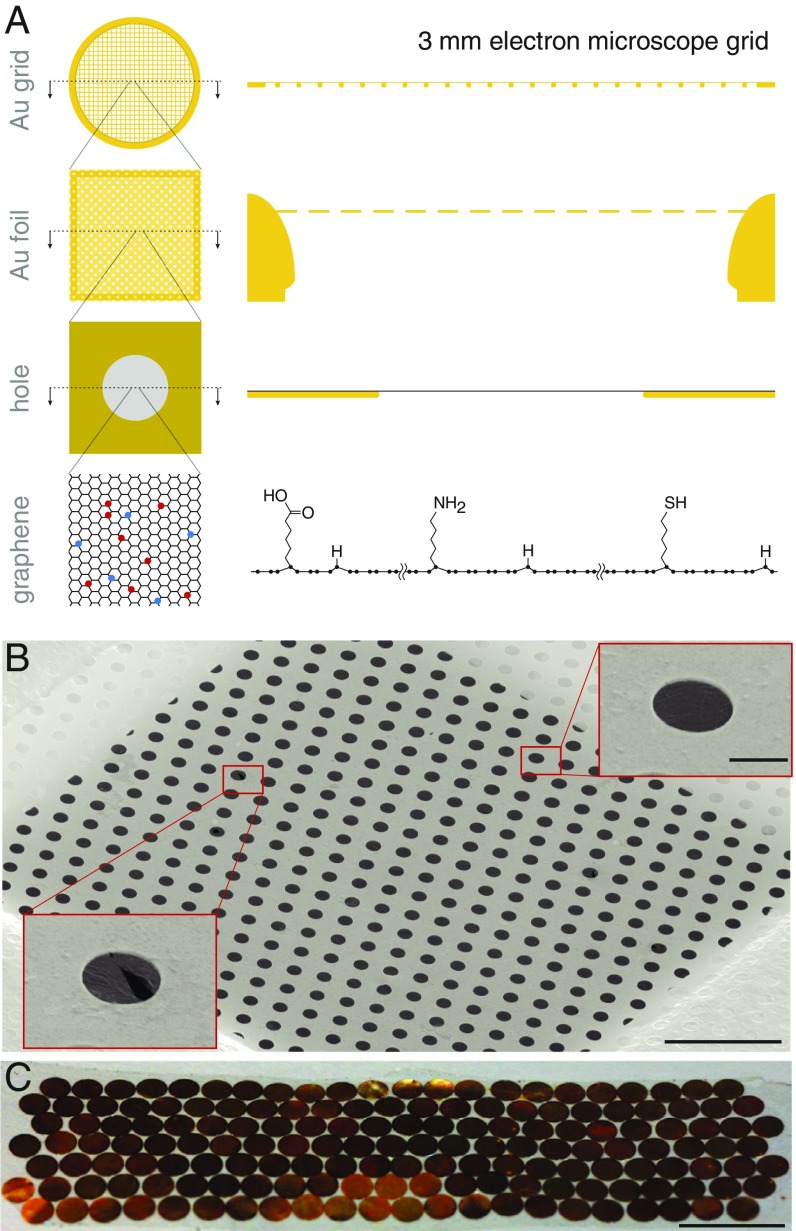
Multifunctional and ultrastable graphene support design. (*A*) The gold-on-gold support comprises a 3-mm disc of gold mesh with 300 lines per inch where an ≃500-Å-thick gold foil with a regular array of submicron-sized holes is suspended across the square openings in the mesh. A graphene monolayer is deposited on top of the gold foil, covering all holes. The graphene can be covalently functionalized by exposure to a low-energy helium plasma containing hydrocarbon precursor molecules (red and blue circles represent different functional groups). The specimen is applied to the graphene side of the grid and is vitrified to form a continuous ice layer which can be imaged through the holes. (*B*) A grid square is covered by the suspended graphene film (secondary electron micrograph). (Scale bar, 10 μm.) *Insets* show a completely covered hole (*Upper*) and a hole with broken graphene (*Lower*), both magnified 5×. (Scale bar, 1 μm.) (*C*) A single growth and transfer batch yields more than 150 graphene-coated all-gold grids with >95% coverage. (Scale bar, 10 mm.)

High-resolution structural studies by electron cryomicroscopy (cryoEM) require the biological specimen to be embedded in a thin layer of amorphous water ice (1, 2). During preparation and cryoplunging, the specimen is exposed to the surfaces of this thin film of water and thus to potentially detrimental interactions with an air–water interface just before vitrification (1, 3). These interactions can be ameliorated with the aid of a thin film covering the specimen support (4), which provides an adherent surface for the specimen—but usually at the expense of introducing background noise, movement during imaging, contamination, and uncontrolled specimen–surface forces. Monolayer graphene is a near perfect support film since it is a conductive material that is only one atom thick, making it effectively invisible in the resolution range of interest to cryoEM (5–8). Pristine graphene, due to its hydrophobicity, is not a suitable substrate for preparing cryoEM specimens; previous work introduced partial hydrogenation with a low-energy plasma as a way of rendering the graphene hydrophilic without damaging the lattice, making it appropriate for cryoEM (8). Still, partially hydrogenated graphene provides only a single type of adherent surface, which is not sufficient for all possible specimens. To address this problem we developed a method to produce multifunctional and ultrastable graphene supports with tunable surface properties for cryoEM (Fig. 1*A*).

## Results

### Design and Production of Multifunctional Graphene Supports.

We optimized a chemical vapor deposition (CVD) graphene growth process (9) to produce 20-${cm}^{2}$ scale sheets of monolayer graphene where the average crystal was larger than one grid square, ≃50 μm across (*SI Appendix*, Fig. S1). We aimed to reduce the number of graphene crystal grain boundaries in the film to increase the mechanical and chemical robustness during subsequent functionalization and imaging. To combine the stability of all-gold specimen supports (10) with the benefits of a controllable graphene surface (8), we devised a clean and scalable graphene transfer procedure (Fig. 1 *B* and *C*). This method preserves both the structural integrity and tensile strength of the suspended gold foils, covering them with a continuous monolayer film of large graphene crystals, while minimizing contamination of the graphene surface (*SI Appendix*, Figs. S2–S4).

**Fig. 2.**
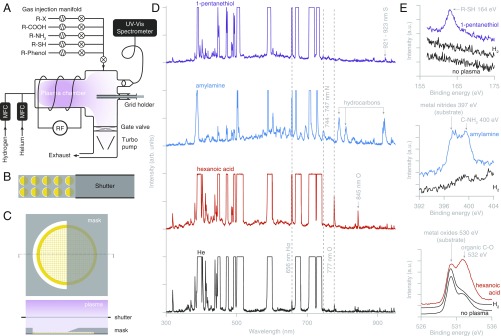
Covalent graphene functionalization and patterning with a low-energy helium plasma. (*A*) Design of an inductively coupled plasma instrument for covalent functionalization of graphene. (*B*) The slotted grid holder positions reliably and precisely up to 10 grids under a single-mask plate. The retractable shutter is used to control the exposure time independent of establishing a stable plasma reaction condition in the chamber. (*C*) A noncontact knife-edge mask design for patterning of graphene grids with plasma-assisted functionalizations. Masks with arbitrary shapes are possible. (*D*) Optical spectra of pure helium carrier plasma (black) and with introduced vapors of chemicals for functionalization. The He spectral lines are saturated to make the other peaks, corresponding to the atomic species from the precursors, visible. The Hα spectral line at 656 nm is used to monitor the breakdown of the hydrocarbon precursor in real time. (*E*) XPS spectra of functionalized graphene showing chemical modifications.

We developed an instrument for creating a range of covalent functionalizations of graphene using a low-energy helium plasma. The instrument comprises an inductively coupled plasma generator, attached to a high-vacuum specimen chamber, where vapors of chemical precursors for graphene functionalization are introduced at a controlled rate (Fig. 2*A*). By using helium (which is inert and has a low atomic number) as the primary plasma gas with a remote plasma generator, sputter damage and other changes to the graphene are prevented while chemical reactions between the graphene and the chemical precursors are enabled. This is in contrast to typical residual air glow discharge systems used for cryoEM which create a plasma with sufficient energy to destroy the suspended monolayer graphene even with short exposures (*SI Appendix*, Fig. S5). We tested four precursor chemicals with different terminal reactive groups (*SI Appendix*, Table S1); in principle this method of functionalization can be used with any small-molecule compound provided it has a vapor pressure sufficient to inject into the chamber. We note that the orientation and length of the functional groups are not specifically controlled by this technique, implying there will possibly be a mixture of functional fragments on the surface. Similar plasma-assisted chemical modification and deposition methods are often used in the semiconductor manufacturing industry (11). To create reproducible and well-defined surface reaction conditions we monitored the atomic composition of the plasma in real time via optical spectroscopy (Fig. 2*D*).

The covalent functionalization of the graphene was confirmed by X-ray photoelectron spectroscopy (Fig. 2*E*). An initial hydrogen plasma treatment (8) allowed for subsequent functionalization of the graphene surface with potentially hydrophobic compounds while preserving the macroscopic surface wetting necessary for the formation of a thin ice layer (*SI Appendix*, Fig. S6). The integrity of the graphene lattice was preserved after these treatments, as demonstrated by electron diffraction (*SI Appendix*, Fig. S4); the graphene lattice is also directly visible in electron micrographs at suitably high magnification (*SI Appendix*, Fig. S7). The surface properties of functionalized grids are preserved when stored in vacuum; contamination in air occurs in 1–10 h, depending on the storage vessel (*SI Appendix*, Fig. S8).

We also designed a noncontact method to pattern multiple functionalizations across the graphene surface of a 3-mm support (Fig. 2 *B* and *C*), allowing one to simultaneously test multiple conditions for the same biological specimen on a single grid. Multiple exposures allow multiaxis patterning (*SI Appendix*, Fig. S6) and serial patterning of the same region with multiple chemicals. At the typical plasma process pressure of ∼1 Torr, and with ≃50-μm mask-to-surface spacing, the resolution of this patterning method is ∼50 μm or 1 grid square (*SI Appendix*, Fig. S5*A*). With more complicated masks, a distinct functionalization for each of the hundreds of grid squares on a grid is possible; smoother surface patterning transitions or gradient functionalization can also be achieved by increasing the distance between the mask and the graphene surface. This patterning method also enables us to selectively deposit amorphous carbon onto a 100-μm-diameter region in the center of the graphene-coated gold grid to allow Thon ring-based optical alignments on the same multifunctional grid before high-resolution data collection (*SI Appendix*, Fig. S9).

### Control of Specimen–Surface Interactions on Multifunctional Graphene Supports.

**Fig. 3.**
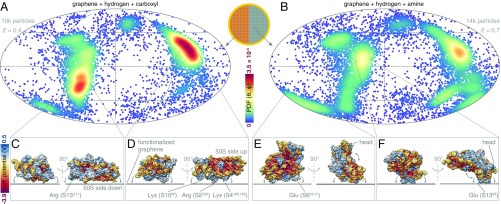
Mapping the surface interaction profile of the 30S ribosomal subunit on a multifunctional graphene grid. (*A* and *B*) The orientation distributions of 30S ribosomal subunits on two different graphene functionalizations from a single patterned grid are plotted on a Mollweide projection. Each point represents an observed particle and the color scale represents the calculated normalized probability density of observing a particle in a particular orientation. E is the efficiency of each orientation distribution (14). The directions of the two most probable views and the variations around these are labeled. (*C*–*F*) The surface of the 30S ribosomal subunit is colored by Coulombic electrostatic potential. *Insets* show the particle orientation on the graphene sheet for each of the labeled views. The arrows show the rotations of the particle on the surface around this view. The in-plane rotation angle is chosen to show the directions with the largest and smallest spread for each view.

We illustrate the potential of multifunctional graphene supports for optimizing the specimen orientation distribution using the 30S ribosomal subunit as a test specimen (12, 13). We prepared graphene functionalized with amylamine, hexanoic acid, 1-pentanethiol, and 4-pentylphenol by applying each of these chemicals to one-half of a partially hydrogenated graphene-coated gold grid. We analyzed the orientation distribution of the 30S particles on each of these surfaces (Fig. 3 *A* and *B* and *SI Appendix*, Fig. S10 *A–D*). We also compared these distributions to those on partially hydrogenated graphene and in unsupported ice (*SI Appendix*, Fig. S10 *E* and *F*). We were able to identify a combination of functionalized surfaces which increased the efficiency of the orientation distribution (14) to 0.8, enabling us to calculate a high-resolution reconstruction of the 30S subunit alone (*SI Appendix*, Fig. S11). The clear differences between all observed distributions indicate the particles are interacting with the functionalized graphene; a tomographic reconstruction shows that the particles are in a monolayer on the graphene surface in a layer of ice just thicker than the particle diameter (*SI Appendix*, Fig. S12 and Movie S1) (15).

Interestingly, these experiments reveal a functional interaction map of the surface of the 30S ribosomal subunit. This allows us to create a physical model of the particle–surface interaction which accounts for the observed orientation distributions. On the carboxylated support, the dominant view indicates that the protein-rich exterior side of the 30S faces the surface (Fig. 3*D*). Several positively charged amino acid residues, including Arg-${S2}^{226}$, Lys-${S10}^{80}$, and Lys-${S4}^{166,169}$, are exposed on this side and likely stabilize contact with the surface. The same orientation is more favored on the thiol-functionalized surface (*SI Appendix*, Fig. S10*B*), consistent with the low pK_a_ of a thiol group. The next most frequent orientation on the carboxylated graphene is with the 50S side of the 30S subunit facing the surface (Fig. 3*C*). The interactions that cause this orientation are likely similar to the ones occurring at the 30S–50S interface in vivo. The putative anchor points include the Arg/Lys-rich S13 chain near the head of the 30S and the 16S rRNA 5′ domain at the surface of the body. This orientation is strongly favored on partially hydrogenated graphene (*SI Appendix*, Fig. S10*E*). In contrast, it is underrepresented on amylamine-treated partially hydrogenated graphene which is consistent with the high pK_a_ of an amine group. Of all surfaces tested here, the amylamine/hydrogen-functionalized graphene minimized orientation bias for the specimens tested. Still, some acidic amino acid residues (Glu-${S6}^{24,31}$, Glu-${S13}^{58}$) can be identified as putative interaction points of the 30S subunit contacting the amine–graphene surface (Fig. 3 *E* and *F*). The Debye screening length in the buffer used is ∼10 Å, and therefore we consider only electrostatic interactions with amino acid side chains that come to closer contact with the graphene surface. The spread of views around each preferred direction is determined by the shape of the particle and how it limits rotation around the fixed interaction point.

### High-Resolution Structure Determination on Multifunctional Graphene Supports.

**Fig. 4.**
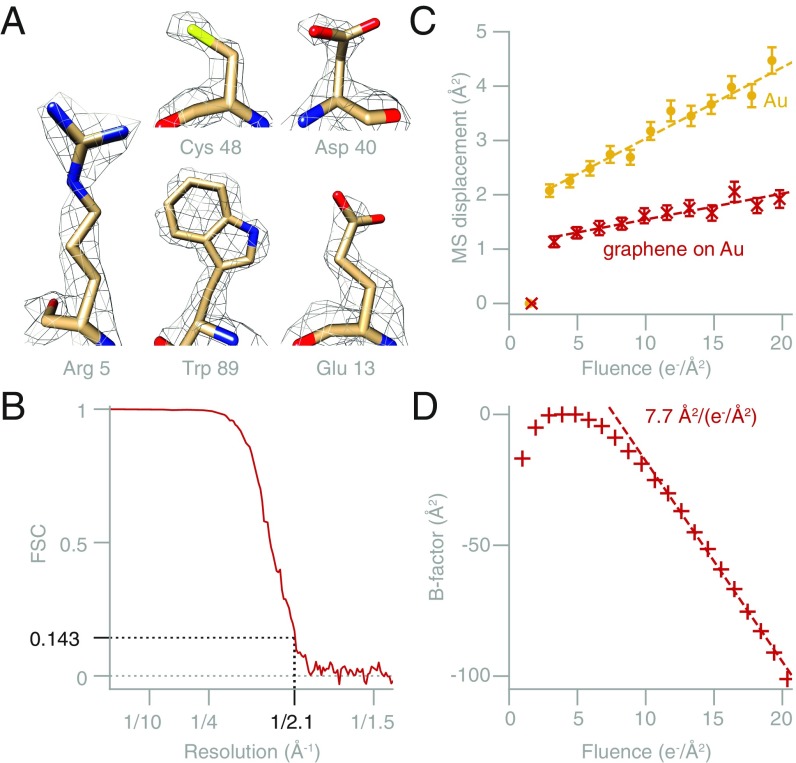
High-resolution structure determination using multifunctional graphene-on-gold supports. (*A*) Contoured density maps of apoferritin, showing amino acid side chains within the structure. (*B*) Fourier shell correlation (FSC) plot for the apoferritin structure. (*C*) Mean-squared displacement of gold nanoparticles unsupported in ice (yellow circles) or in ice on graphene (red crosses), on all-gold supports. The addition of the graphene film reduces both the movement at the beginning of irradiation and the diffusion-like movement during the later frames by 2×. The dashed lines are linear fits to the data excluding the first frame. The slope decreases from 0.13 ± 0.1 Å^2^/(e^−^/Å^2^) to 0.048 ± 0.005 Å^2^/(e^−^/Å^2^) with the addition of the graphene to the all-gold specimen support. (*D*) Calculated B-factors as a function of electron fluence for the apoferritin dataset. The B-factor in the first 3 e^−^/Å^2^ corresponds to the initial nondiffusional movement phase. The B-factor decreases at an approximately constant rate of 7.7 ± 0.8 Å^2^/(e^−^/Å^2^) with fluence in the range 10–20 e^−^/Å^2^ (dashed line shows linear fit).

To demonstrate that these supports are suitable for high-resolution structure determination, we used an amylamine-functionalized graphene on gold support to determine the structure of horse spleen apoferritin. The reconstruction reached 2.1-Å resolution (0.143 Fourier shell correlation [FSC] (16)) from 41,202 particles with standard data collection and processing (*SI Appendix*, Fig. S7 and Table S2 and Fig. 4*B*). The resolution of the reconstructed map, which is equivalent to the highest reported to date for this specimen (17), but required about half as many data, is demonstrated by clear densities for side chains in the map (Fig. 4*A*). This includes complete aspartate and glutamate residues, for which densities beyond the Cβ are often absent from EM maps (18). The decay rate of high-resolution information with fluence after the first 5–10 e^−^/Å^2^ of irradiation in this dataset (Fig. 4*D*) is similar to measurements of radiation damage in 2D protein crystals (19, 20). We compare the movement of particles on multifunctional graphene-on-gold supports to previous measurements on several other supports (*SI Appendix*, Figs. S13 and S14) (4). Adding a graphene layer to an all-gold support reduced particle movement by a factor of 2 during electron irradiation. Compared with graphene-on-carbon supports, graphene-on-gold supports reduced particle movement by a factor of 3. The reduction in particle motion was also verified by tracking the movement of individual gold nanoparticles on an all-gold support with and without an additional graphene layer (Fig. 4*C* and *SI Appendix*, Fig. S15). Importantly, besides reducing the rate of random (uncorrelated) particle motion, the addition of the graphene layer also reduces the movement during the first few electrons of irradiation, improving the quality (*B*-factor) of these initial frames (Fig. 4 *C* and *D*), in which the molecules are less damaged by the beam.

## Discussion

Multifunctional graphene-on-gold supports provide several improvements over previously described graphene oxide and graphene-on-carbon supports (6, 8, 21, 22). The graphene growth, transfer onto all-gold grids, functionalization, and patterning methods presented here are all scalable; more than 100 grids can be processed in one day and this could be easily increased for commercial manufacture. Besides being a tunable surface for improving specimen orientation in cryoEM, functionalized graphene provides a method of mapping and quantifying the interaction surface of a particular biomolecule. Knowledge of how a purified protein interacts with other well-defined surfaces, like those present in ion exchange, hydrophobic interaction, or other affinity purification columns, will help guide the choice of appropriate functionalization and buffer conditions for a particular cryoEM specimen. This includes small proteins which would benefit from the reduced background signal and movement on graphene and membrane-bound molecules prepared by a range of solubilization and purification methods. With the spatial resolution of the noncontact plasma patterning method described here, grids on which every square has a distinct functionalization and surface property or linker chemistry are possible. Reactive chemical groups bound to the graphene surface, in particular thiol groups, can serve as a versatile platform for further covalent modifications, including to display ligands for specific binding on short flexible hydrocarbon linkers.

Graphene-on-gold specimen supports reduce the motion of the specimen, improving electron micrograph quality. We posit that the twofold reduction in the initial movement of the particles in ice is due to the graphene layer restricting the bulk movement of the thin film of ice within each hole. This occurs as mechanical stress—likely caused by the differential contraction of the gold foil and the water during vitrification—is released at the initiation of irradiation with the electron beam. This bulk movement of ice is evident from the correlation between the trajectories of neighboring particles in the first e^−^/Å^2^ (*SI Appendix*, Fig. S15 *B* and *E*), which rapidly become uncorrelated. The reduction of the movement in the second, uncorrelated phase may be due to a reduction in the degrees of freedom of the particle adhered to the graphene surface. Still, complete elimination of the initial movement of the specimen would improve the images even further, reducing the movement to the limit set by pseudodiffusion of the particles in ice (23) (*SI Appendix*, Fig. S14 *E* and *F*). One strategy to reach the pseudodiffusion limit is to improve motion correction algorithms by using the graphene lattice as a fiducial, as suggested previously for graphene oxide (24). This approach is now more likely to succeed when starting from the sub-2-Å movement afforded by this specimen support design, but still relies on the movement of the lattice and the particle being correlated. We have made an entire raw dataset publicly available via the EMPIAR database to allow others to improve data-processing algorithms for micrographs on ultrastable graphene supports. We envision that patterned, multifunctional graphene-on-gold supports will be instrumental both in rapidly finding optimal surface conditions for cryoEM specimen preparation with a minimum of effort and microscope use and in determining high-resolution structures with the fewest possible data.

## Materials and Methods

### Graphene Growth and Transfer.

#### Chemical vapor deposition of graphene on copper.

Graphene was grown on copper foil (Alfa Aesar 46365) in our in-house–constructed graphene CVD system with a 25-mm-diameter reaction tube. Copper foil was cut to 65-mm × 23-mm strips and cleaned by exposing each side to 120 s of UV-Ozone, before submersion into 200 mL 20% hydrochloric acid, followed by washing in deionized 18 M$\Omega \;H_{2}$O (Millipore) and drying with nitrogen gas. The copper foil was then placed in the quartz tube of the CVD system under flow of argon and heated to $1,050{}^{○}$C for annealing, during which the copper grain size increased from ∼10 μm to ∼1 cm scale. This was followed by annealing under hydrogen flow at 20 sccm and pressure 0.3 Torr for 1.5 h. At the end of the hydrogen anneal, oxygen gas (BOC, N6 purity) was applied for 120 s at pressure of 1.8 mTorr (9). After this, graphene was grown at $1,035{}^{○}$C under a flow of 0.072 sccm methane, 20 sccm hydrogen, and 30 sccm argon, at total pressure of 500 mTorr for a duration of 8 h. Typical growth conditions for the graphene used in this work are shown in *SI Appendix*, Fig. S1*A*. During the postgrowth cool down, oxidation was applied at $180{}^{○}$C for 1 h to make the graphene-coated copper regions easily distinguishable from the uncoated ones. By varying the graphene growth conditions and increasing the growth time, we could in principle produce grains as large as 1 mm and control the extent of coverage of the substrate. The target crystal size was chosen at 100 μm, sufficient to cover an entire grid square while minimizing growth time.

#### Graphene transfer to all-gold grids.

Graphene was transferred onto all-gold grids (UltrAuFoil R0.6/1; Quantifoil) using a compliant form of collodion polymer. This method (*SI Appendix*, Fig. S2*A*) was chosen after many tests since it preserves the structural integrity of the gold foil, while keeping the large graphene sheet intact, and provides optimal plastic adhesion for the transfer, conformal coating of the surface of the grid, and subsequent removal of the polymer. Approximately 100 μL collodion solution (2% cellulose nitrate in amyl acetate; Sigma) was pipetted onto the meniscus of a 135-mm-diameter crystallization dish filled with deionized 18 M$\Omega \;H_{2}$O, and the graphene-covered copper was gently placed on top. Within 5 min, as the solvent evaporated, the collodion solidified and the foil could be picked up. Any excess collodion around the foil area was removed, and the foil was turned over (with the collodion side up) and placed flat on the surface of the copper etchant (${FeCl}_{3}$ based; Sigma) in another 135-mm-diameter crystallization dish. The etchant was partially drained and the dish was refilled with 100 mL 20% HCl; this wash was usually repeated 10 times. This was followed by 5 washes with 2% HCl, 5 washes with 0.2% HCl, and 10 washes with deionized 18 MΩ water. Then the water was siphoned out and the collodion-supported graphene was lowered onto the batch of all-gold grids, arranged on the bottom of the dish. From one copper foil we could obtain 150–200 graphene-coated all-gold grids in a single transfer. The collodion was removed from the grids before use by immersing each grid in amyl acetate, 2-ethoxyethanol, chloroform, acetone, and isopropanol (all high-purity semiconductor grade; Sigma) in this order.

#### Graphene quality control.

Examination by eye, optical microscopy, and scanning electron microscopy was applied to monitor the degree of graphene growth and surface contamination. Scanning electron images of graphene on copper and suspended graphene were acquired on a Scios DualBeam FEG SEM (FEI) in secondary electron mode using an Everhart–Thornley detector (*SI Appendix*, Figs. S1*C*, S2*B*, and S3). Transmission electron diffraction experiments were performed on the suspended graphene to assess the structure of the crystals. Weak and monotonic variation of the intensity of the graphene diffraction spots with tilt unambiguously identified suspended monolayer graphene (*SI Appendix*, Fig. S4 *A* and *B*) (25). The mean linear intercept grain size of the graphene was measured to be 60 ± 10 μm (*SI Appendix*, Fig. S4*C*), meaning one could expect to encounter only one to two grain boundaries per grid square. For this measurement the number of different grain orientations observed in the selected-area electron diffraction pattern was counted while traversing the whole grid in the TEM. We and others have previously characterized the background signal of graphene and hydrogenated graphene vs. thin layers of amorphous carbon and graphene oxide (7, 8). We have also measured the background signal in power spectra from grids transferred and functionalized as reported here and found it was 6–7× less than for a 46-Å-thick layer of amorphous carbon, whose thickness was measured accurately by atomic force microscopy. Thus, the background signal is slightly more than that of pristine graphene (3-Å-thick crystalline lattice) but roughly equivalent to 6 Å of amorphous carbon. We note that residual polymers, including nitrocellulose, left on the graphene can align with the lattice and can be observed in the low-dose diffraction pattern, as reported previously for PMMA (26). We observed this as well when the collodion was not completely removed from the graphene layer. Still, unlike for PMMA, for collodion this residual contamination can be avoided through the careful use of semiconductor grade solvents and the plasma treatment method described here.

### Graphene Functionalization.

#### Covalent functionalization using a low-energy helium carrier plasma.

Plasma treatments were performed in an extensively modified commercial plasma cleaner (Fischione 1070), equipped with a custom grid and mask holder, a custom gas-injection system (Fig. 2*A*), and a custom fiber-coupled sapphire viewport attached to a UV-vis spectrometer (Thorlabs). The vacuum chamber was evacuated to $<10^{-5}$ Torr by a turbo pump. A carrier plasma of N6.0 grade helium was produced at 28 sccm flow rate and typical pressure $5\times 10^{-1}$ Torr. The 18-MHz RF coil was operated at 40% power, yielding typically 9 W forward power and 0.6 W reverse power. The vapors of the chemicals used for functionalizations were introduced through an evacuated five-channel manifold. The chemicals were stored in sealed, evacuated 1-mL stainless steel vials, and the vapor of the desired chemical was supplied through a precision micrometer needle valve and a shutoff valve into the plasma chamber. The effect of the introduced chemical was observed in the real-time spectrum of the plasma. Spectra were acquired with a 6-s exposure time, which saturated the helium lines and made the additional peaks from the precursors visible. The most prominent feature in the optical spectra due to the introduction of organic molecules was the Hα peak at 656 nm (Fig. 2*D*). We hypothesize that this is due to the high probability of liberating terminal hydrogens from the molecule; whereas the separation of larger fragments or nonterminal moieties, like the atomic sulfur from a thiol, would require two or more bonds to be broken, and therefore is less likely. The actual amount of chemical present in the plasma depends on the vapor pressure of each compound; this can be adjusted by controlling the temperature of the liquid container and the setting on the leak valve. Once the desired plasma composition was established, a shutter covering the grid and mask holder was used to control the exposure time. Between the uses of different chemicals, the chamber was cleaned using a pure helium plasma at 70% power for 30–60 min, until no signs of trace hydrogen were visible in the optical spectrum.

#### X-ray photoelectron spectroscopy of functionalized graphene.

X-ray photoelectron spectroscopy (XPS) was used to study the covalent modifications to graphene. The beam from an Al Kα source (1,486.68 eV; ESCALAB 250 Xi) was focused in a 900-μm-diameter probe on the sample. Graphene on copper (as grown) and graphene transferred to UltrAuFoil gold grids were used for these measurements, and charge compensation was applied. Partially hydrogenated and nontreated graphene specimens were used as controls to compare against functionalized graphene. The pressure in the XPS chamber during data acquisition was 5 $\times 10^{-9}$ mbar. First, spectral scans at 1 eV sampling were acquired in the 136- to 1,361-eV range with 200-eV pass energy, and then 30–50 scans with 30- to 50-eV pass energy at 0.1-eV sampling were acquired around the regions of interest and averaged to produce the spectra plotted in Fig. 2*E*. The peaks were identified using data from the NIST XPS Database (27). Some of the observed peaks were attributed to signal from the metal substrate supporting the graphene.

#### Contact angle measurements.

Contact angle measurements were performed to evaluate the surface properties of the support after varying doses of functionalization (*SI Appendix*, Fig. S6*F*) and to determine the effect of various storage conditions on these properties over time (*SI Appendix*, Fig. S8). We used a custom instrument to measure the contact angle of pure water on the grids (28). Briefly, a 1- to 2-μL droplet of deionized 18 MΩ water was pipetted to the center of the clamped grid and imaged parallel to the surface against a background of diffuse illumination. Any images where the grid appeared bent or the droplet spread asymmetrically were discarded. The typical time between the end of the plasma treatment and the measurement was 1 min; for each data point three to five repeats were performed. The contact angle was quantified in ImageJ (29). To demonstrate the stability of the surface modification when stored in vacuum, we treated a graphene-coated gold grid with 10 min hydrogen plasma and stored it at $<10^{-5}$ Torr for 15 h. We found the contact angle to be $60\pm 2^{○}$ after this storage compared with $57\pm 3^{○}$ immediately after treatment. This is in contrast to grids stored in air in a variety of typical conditions, including plastic grid boxes and glass Petri dishes (*SI Appendix*, Fig. S8) which become contaminated and lose their hydrophilicity within 1–10 h. Further work is needed to provide a simple and commercially viable long-term storage container for functionalized grids; a sealed, evacuated glass vial similar to those used for storing chemicals is one possibility.

#### Patterning of functionalized graphene.

To pattern grids with multiple functional groups in different regions of each grid, a knife-edge mask was precisely positioned above the top surface of each grid for each plasma processing step (Fig. 2*C*). This was done using a set of precision-machined masks that fit in a defined position above a grid alignment holder. The apparatus was capable of patterning 2 × 10 grids at a time (Fig. 2*B*). The sharpness of the pattern depends on and can be controlled by varying the following: the distance between the mask and the graphene surface, the sharpness of the mask edge, and the mean free path of the species in the plasma. Under the typical conditions used here (1 Torr, nonthermal remote plasma), the mean free path in the chamber is much larger than the other relevant length scales; we therefore estimate the patterning resolution to be approximately equal to twice the distance between the grid and the mask, i.e., 50 μm. This is confirmed by the width of the transition region in *SI Appendix*, Fig. S5*A* for a knife-edge mask placed 20 μm above the surface of the foil.

#### Amorphous carbon deposition onto graphene-on-gold supports.

Amorphous carbon was deposited directly onto graphene-coated gold grids in an Edwards 306A evaporator evacuated to $10^{-6}$ mbar. The grids were positioned in a custom slotted holder, analogous to the one used for plasma treatments, and covered by a mask plate. Single-slot apertures with 100-μm hole diameter (EMS GA100-Au) were used for masks to expose only the center of the grid to the evaporated carbon. The grid-to-mask distance was ∼100 μm. The localized deposition of a continuous amorphous carbon film on the graphene was verified by TEM imaging (*SI Appendix*, Fig. S9).

### Motion Tracking in Vitreous Ice Using Gold Nanoparticles.

#### Grid preparation.

Specimens were prepared by manual plunge freezing in a $4{}^{○}$C cold room. All-gold supports (UltrAuFoil R 0.6/1, 300-mesh; Quantifoil) with 800-nm hole diameter, with and without graphene, were used. Specimen supports were plasma treated to render them sufficiently hydrophilic before the vitrification as follows: 60 s 9:1 Ar:$O_{2}$ plasma for the all-gold grids and 60 s hydrogen plasma for the graphene-coated grids. Then 3 μL of 10-nm unconjugated gold colloid solution at OD 100 (BBI Solutions) were applied to the foil side of the grids and blotted for 15 s from the same side before plunging into liquid ethane held at 93 K (30).

#### Imaging.

The data for the graphene-supported ice were collected on a Titan Krios and for the unsupported ice on a Tecnai Polara, using a Falcon 3 detector in integrating mode in both cases and imaging conditions, typical for single-particle data collection (300 keV, 1 e^−^/pixel per frame, 80 K specimen temperature). The illumination was centered over each exposed hole and the beam diameter was set to be slightly larger than the hole to minimize beam-induced motion (31). The magnification was chosen to include the edges of the hole in the micrograph for drift tracking. This corresponded to 1.74 Å/pixel on the Krios (47,000× nominal) and 1.72 Å/pixel (59,000× nominal) on the Polara. Movies were acquired for a duration of 5–8 s to a cumulative fluence of 60 e^−^/Å^2^. Only holes from squares without defects in the gold foil were imaged.

#### Motion analysis.

The whole-micrograph movies were motion corrected in MotionCorr (32) to remove stage drift. The motion-corrected stacks were binned to give 1.5 e^−^/Å^2^ fluence per frame. Particles were manually picked in EMAN2 (33) and extracted from the motion-corrected stack in 128 × 128-pixel frames. Each particle movie was then motion corrected again in MotionCorr. The corrected-particle movies were visually inspected. The trajectories of all particles during the first 20 e^−^/Å^2^ of irradiation were used to calculate the mean-squared and root-mean-squared displacements of the particles over the ensemble.

### CryoEM of *Thermus thermophilus* 30S Ribosomal Subunit.

#### Grid preparation.

Purified *Thermus thermophilus* 30S ribosomal subunits (in 5 mM Hepes, 50 mM KCl, 10 mM ${NH}_{4}$Cl, 10 mM Mg(OAc)_2_) were provided by the Ramakrishnan laboratory. The concentration was adjusted to 8 mg/mL, except for the specimen on partially hydrogenated graphene, where the concentration was 1.7 mg/mL. All graphene-coated grids used were first exposed to $H_{2}$ plasma for 180 s to render the whole surface hydrophilic, followed by a 30-s treatment of half of the grid with He plasma carrying the vapor of the corresponding functionalizing chemical and another 30-s treatment for the other half. Plain UltrAuFoil grids for the control experiment without graphene were treated with a 9:1 Ar:$O_{2}$ plasma mixture for 60 s. Grids were plunged using an FEI Vitrobot equilibrated at $4{}^{○}$C and 100% relative humidity; the liquid ethane was kept at a fixed temperature of 93 K. A 3- to 4-μL volume of the protein solution was pipetted onto the graphene-coated side of the grid, double-blotted for 5 s, and immediately plunged into the ethane. Typically less than 10 min elapsed between the plasma treatment and the vitrification. The grids were stored in liquid nitrogen until they were transferred into the electron microscope for imaging.

#### Imaging and data collection.

Micrographs of 30S ribosomes in ice on functionalized graphene were acquired on a Tecnai Polara microscope operated at 300 kV using a Falcon 3 detector in integrating mode. The nominal magnification was 93,000×, corresponding to 1.17 Å/pixel (calibrated using the 2.13-Å graphene reflections). Micrographs of 30S ribosome in unsupported ice and in ice on partially hydrogenated graphene were acquired on a Titan Krios microscope operated at 300 kV using a Falcon 3 detector in integrating mode, at nominal magnification 59,000×, corresponding to 1.34 Å/pixel.

#### Single-particle data analysis.

The sectors of the functionalized grids were identified using the asymmetric center mark (*SI Appendix*, Fig. S6*E*). The micrographs were motion corrected in Relion 3 (17), CTFs were fitted using Gctf (34), particles were manually picked in EMAN2, and 2D classification and 3D refinement were done in Relion 3. All particles from the functionalized grids were processed together and the final orientation angles were sorted into categories for each surface based on the micrograph name and the corresponding coordinate on the grid. The particles from the control experiments were processed separately due to differing pixel sizes. The orientation distributions of the particles were analyzed using cryoEF (14) and plotted on an equal-area Mollweide projection, with the color scale representing the local Gaussian kernel density (i.e., probability distribution function [PDF]) of the distribution at every sampled orientation (Fig. 3 *A* and *B* and *SI Appendix*, Fig. S11). The bandwidth for the kernel density was set to $5^{○}$. These plots also represent the normalized probability of the particle interacting with the surface in a particular orientation, thereby quantifying the strength of this interaction. We note that some spread in the assigned orientations might be due to local bending of the foil, wrinkling of the graphene, bulging of the ice, or bending of the flexible parts of the ribosomal subunit, all of which are likely $<5^{○}$. The high-resolution crystal structure of the 30S ribosome (1J5E (12)) was converted to a surface and colored by surface Coulombic potential as calculated in UCSF Chimera (35) to illustrate the dominant orientations of the particle on the graphene and analyze the interaction interfaces (Fig. 3 *C*–*F*). The potential of each orientation distribution to provide uniform Fourier space sampling for enabling high-resolution reconstruction was assessed using the efficiency metric (14). We calculated the efficiencies for each separate orientation distribution from ∼10,000 particles; these can then be used to make a rational decision regarding the most optimal surface or combination of surfaces for further data collection. In this example, the surfaces of choice would be amine- and phenol-functionalized graphene, providing a combined efficiency of 0.8, thereby enabling near-atomic structure determination with ∼100 times fewer particles than, for example, from a dataset of unsupported 30S ribosomes in ice with an efficiency of 0.3 (14) (*SI Appendix*, Fig. S11).

#### Tomography.

Single-axis bidirectional tilt series spanning the −$50^{○}$ to +$50^{○}$ range in $5^{○}$ increments were collected for the same specimen on a Titan Krios operated at 300 keV using a Falcon 3 direct electron detector in integrating mode. The exposure in each image was 2 e^−^/Å^2^ over 1 s (40 frames) at 37,000× nominal magnification. The movies were motion corrected in MotionCorr (32), and the tomogram was aligned without fiducials and reconstructed using SIRT in Etomo (IMOD) (36).

### CryoEM of Human 20S Proteasome.

#### Grid preparation.

A specimen of human 20S proteasome (Enzo) was buffer exchanged into TBE (89 mM Tris, 89 mM boric acid, and 2 mM EDTA) at pH 8.3 and adjusted to 0.8 mg/mL concentration. Four-quadrant grids were prepared by exposing one-half of the grid to 180 s hydrogen plasma and then rotating the mask by $90^{○}$, exposing one-half of the grid to amylamine under helium plasma for 30 s, and rotating the mask by $180{}^{○}$ to expose the other half of the grid to hexanoic acid under helium plasma for 30 s. Vitrification was done in the same way as above.

#### Imaging and data collection.

Micrographs were acquired on a Titan Krios microscope operated at 300 kV using a Falcon 3 detector in integrating mode. The flux was set to 17 e^−^/Å^2^/s and the exposure time was 2 s; a 70-μm objective aperture was used. The nominal magnification was 59,000×, corresponding to 1.34 Å/pixel, calibrated using the (101) reflections from anatase (${TiO}_{2}$) nanoparticles dispersed on a separate calibration grid. The quadrants of the grids were identified with respect to the orientation of the asymmetric grid center mark as above (*SI Appendix*, Fig. S6).

### CryoEM of Apoferritin.

#### Grid preparation.

Horse (*Equus caballus*) spleen apoferritin (Sigma) was buffer exchanged into 100 nM PBS at pH 7.4 and adjusted to 11.7 mg/mL concentration. Graphene-coated grids treated with He plasma carrying amylamine for 30 s were used. Vitrification was done in the same way as above.

#### Imaging and data collection.

Movies were acquired on a Titan Krios microscope operated at 300 kV using a Falcon 3 detector in counting mode. The nominal magnification was 120,000×, with pixel size 0.6495 Å/pixel, calibrated using the 2.347-Å (111) Au reflections in the Fourier transform of a micrograph of the gold support foil taken under the same conditions as used for data collection. A 100-μm objective aperture was used during data collection. Movies were acquired with 30.01 s exposure time, and the frames were grouped into 38 fractions (31 frames per fraction). The flux was set to 0.53 e^−^/pixel/s, corresponding to a total fluence of 37 e^−^/Å^2^ in each movie.

#### Single-particle data analysis and modeling.

The micrographs were motion corrected in Relion 3, CTFs were fitted using Gctf, particles were manually picked in EMAN2, and 2D and 3D classification, 3D refinement, CTF refinement, particle polishing, and postprocessing were all done in Relion 3. The resulting EM density map reached 2.14 Å gold-standard FSC resolution, had an overall B-factor of −54 Å^2^, and was in agreement with the corresponding high-resolution crystal structure (2W0O (37)) (*SI Appendix*, Table S2). The previously published model (4V1W (10)) was fitted as a rigid body in the map reported here using MolRep (38) and refined using RefMac (39), followed by manual editing in Coot (40). Illustrations of the 3D map with the fitted model were rendered in Chimera. The orientation distribution of the particles contributing to the final reconstruction was analyzed using cryoEF and showed an efficiency of 0.7. For the motion analysis, the displacement of each particle throughout the movie was calculated by subtracting the whole-micrograph movement from the particle movement as calculated by Bayesian polishing in Relion (41). This was necessary since the dominant component of the motion was drift of the microscope stage at a rate of 0.6 Å/s throughout the 30-s exposure.

## Supplementary Material

Supplementary File

Supplementary File
