# Supplementary material for: Multifunctional graphene supports for electron cryomicroscopy
Source: Proc Natl Acad Sci U S A. 2019 May 24;116(24):11718–24. doi: 10.1073/pnas.1904766116 (PMC6575631; doi:10.1073/pnas.1904766116)
Supplement: Supplementary File [file pnas.1904766116.sapp.pdf]

Supplementary Information for

Multifunctional graphene supports for electron cryomicroscopy

Katerina Naydenova, Mathew J. Peet and Christopher J. Russo

Corresponding Author: Christopher J. Russo
E-mail: crusso@mrc-lmb.cam.ac.uk

This PDF file includes:

Figs. S1 to S15
Tables S1 to S2
Caption for Movie S1
References for SI reference citations

Other supplementary materials for this manuscript include the following:

Movie S1

www.pnas.org/cgi/doi/10.1073/pnas.1904766116

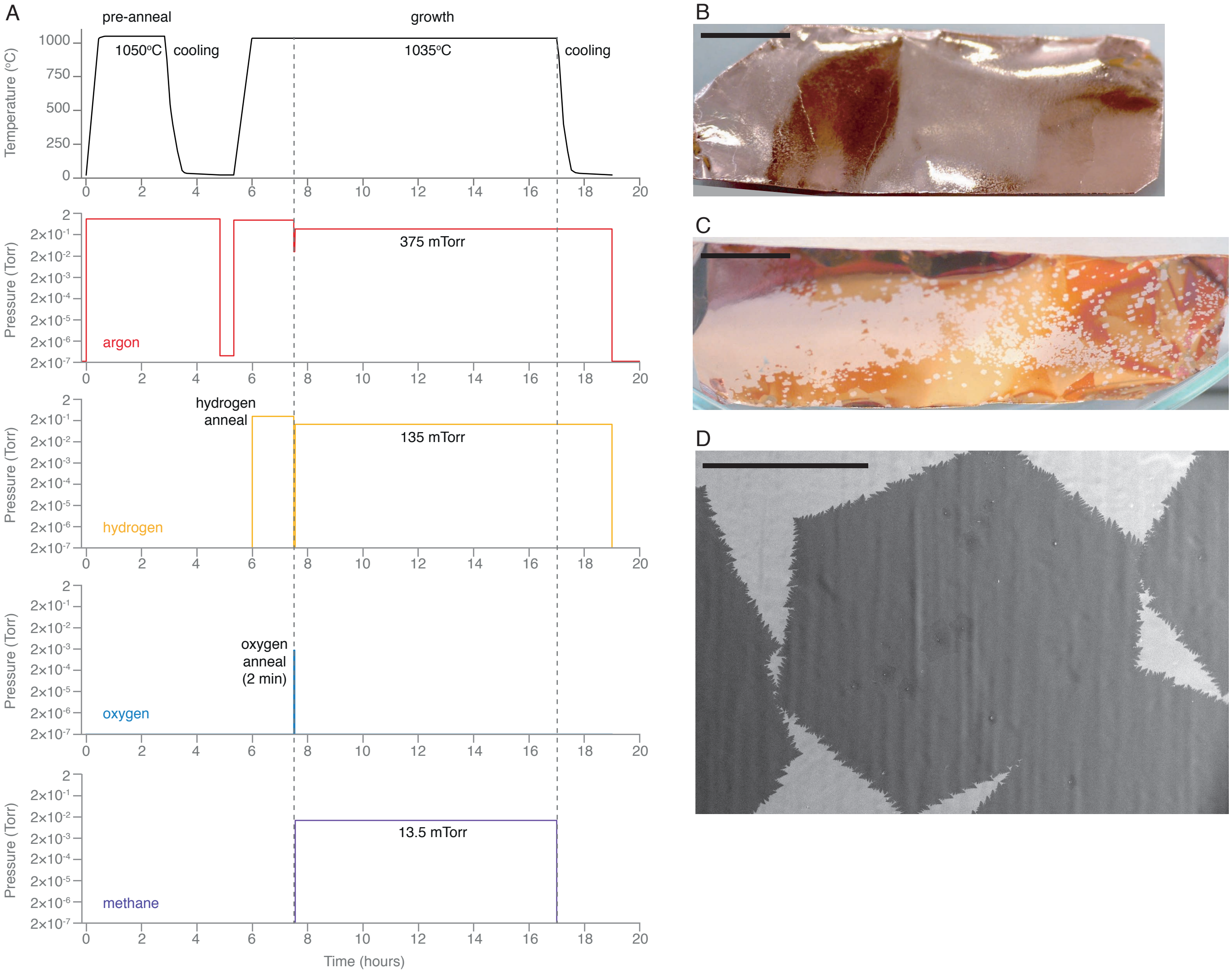

Fig. S1. Graphene growth by chemical vapor deposition. (*A*) Typical temperature and partial pressures of the gases in the CVD chamber during the graphene growth process. (*B*-*C*) Optical micrographs of copper foil after graphene growth and oxidation. The foil in panel (*B*) is fully covered in graphene, therefore the copper surface did not oxidize. The foil in panel (*C*) is only partially covered due to early termination of the growth process. The non-covered copper regions were oxidized and appear red in color. The individual hexagonal graphene crystals have sizes of $\sim$1 mm. Scale bar for (*B*) and (*C*) is 10 mm. (*D*) Scanning electron micrograph of the hexagonal graphene crystals on the copper foil from panel (*C*). Scale bar is 200 μm.

 Katerina Naydenova, Mathew J. Peet and Christopher J. Russo

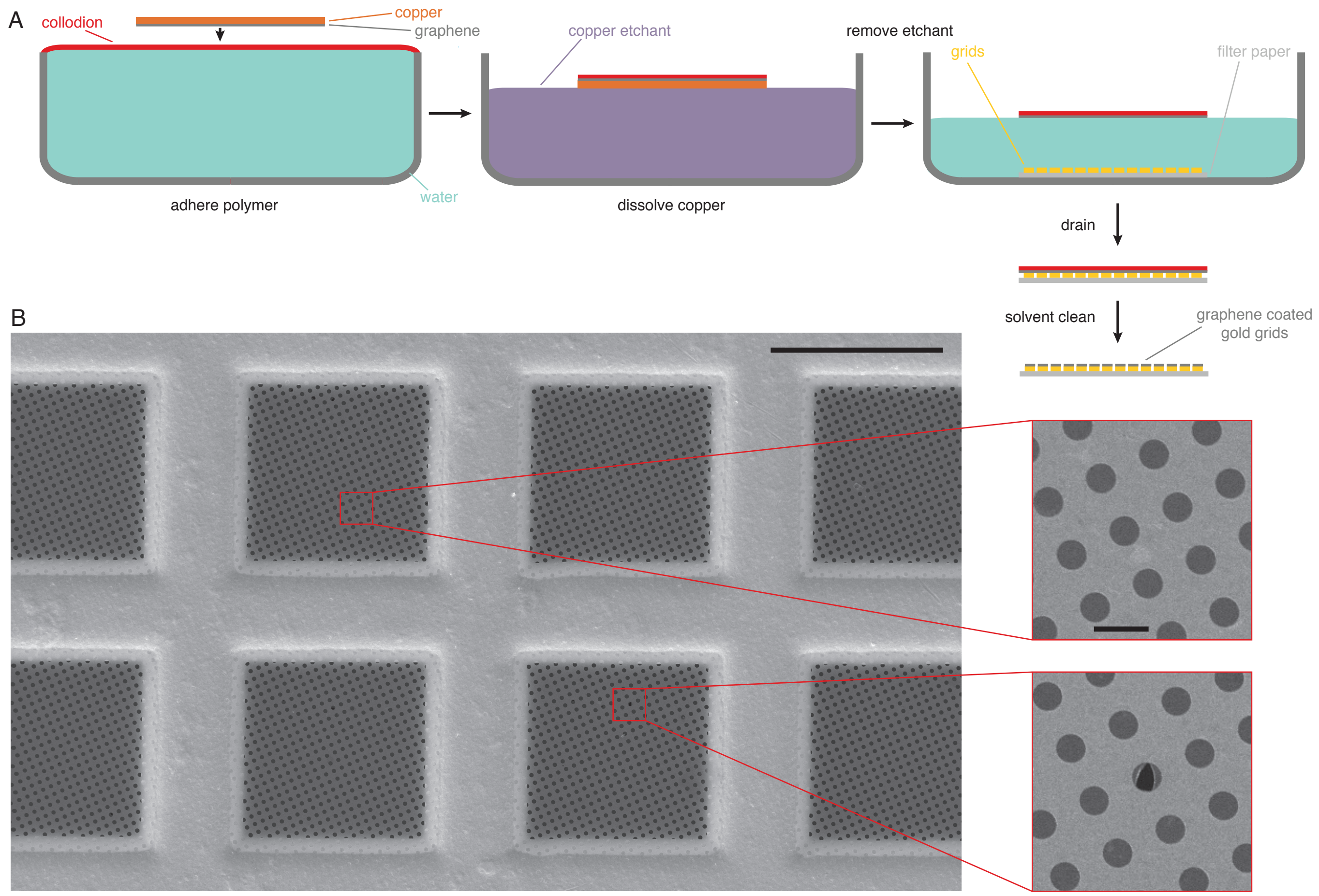

Fig. S2. Scalable graphene transfer process optimized for preserving the structural integrity of the graphene and the all-gold grids. (*A*) Typical collodion-assisted graphene transfer onto all-gold grids (see Methods). (*B*) Representative micrograph of a region on the all-gold grid after the graphene transfer. Approximately 99% of the holes are fully covered with intact graphene. Scale bar is 50 μm. The insets show the squared areas magnified. Scale bar for the insets is 2 μm.

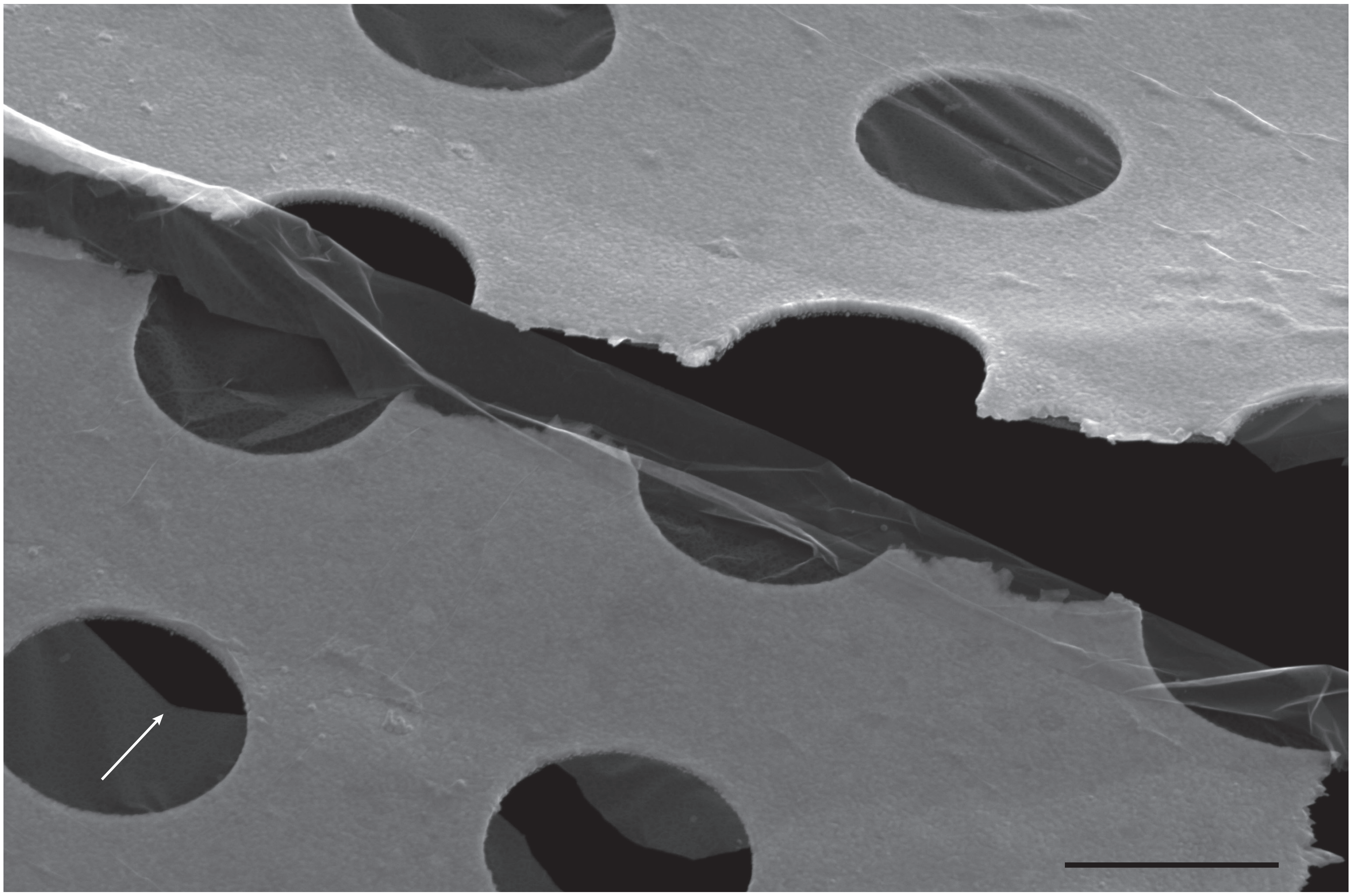

Fig. S3. Structural stability and adhesion of the suspended graphene on holey gold foil. Scanning electron micrograph of suspended graphene on gold foil. The graphene film is robust enough to withstand handling of the grids during sample preparation, as evident from some intact holes adjacent to a broken foil region, and is able to self-support over micrometer distances. When mishandled, the graphene crystal breaks along the crystallographic directions forming the characteristic 120° angle (arrow). Scale bar is 1 μm. The micrograph was acquired using 30 keV electron beam at 0.2 nA current with an ETD detector in secondary electron mode (30 μs dwell time per pixel, 6.9 mm working distance) and the sample was tilted by 60°.

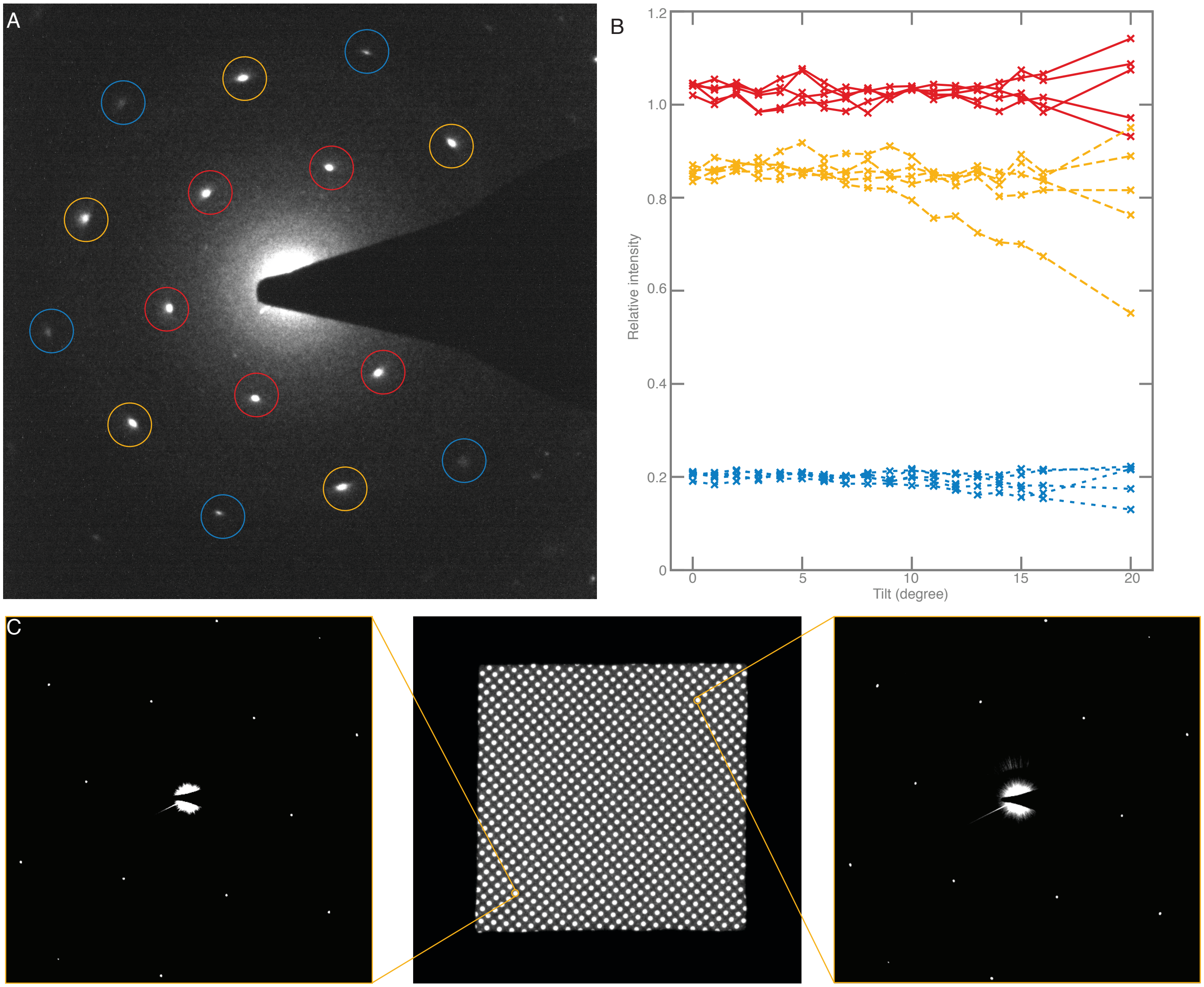

Fig. S4. Demonstration of single-crystal monolayer graphene via electron diffraction. (*A*) Selected-area electron diffraction pattern of monolayer graphene at 0° tilt. The first-order reflections correspond to 2.13 Å. Panel (*B*) shows the variation of the integrated total intensity with tilt for the circled diffraction spots (1). The lack of any significant change in intensity with tilt angle shows that the graphene is one atom thick. (*C*) Selected-area electron diffraction pattern from two holes in the opposite ends of the same grid square show the same graphene crystal extends over distance greater than 50 μm.

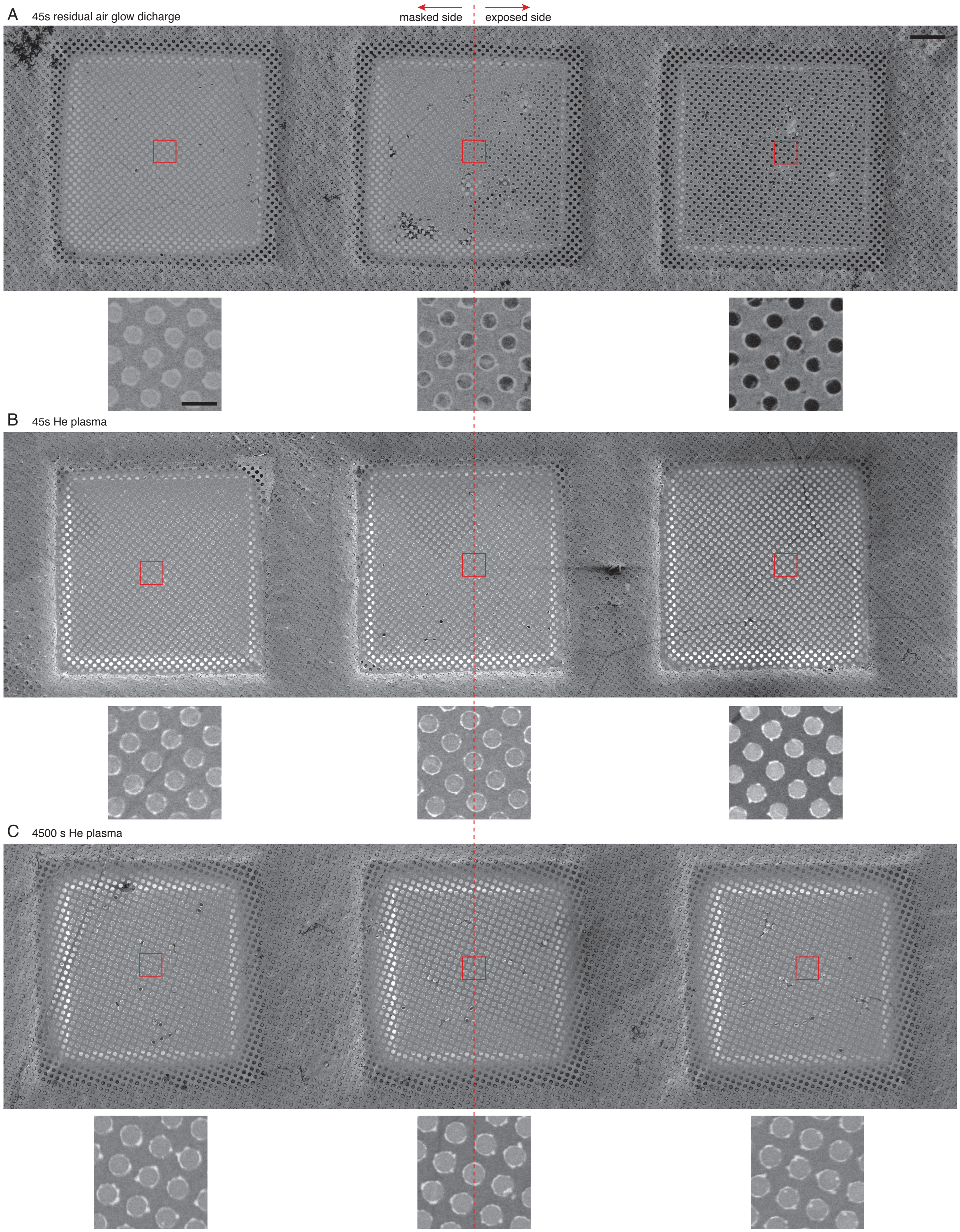
A 45s residual air glow dicharge
masked side
exposed side
B 45s He plasma
C 4500 s He plasma

Fig. S5. Effect of residual air glow discharge and helium plasma on monolayer suspended graphene. (Previous page) Scanning electron micrograph of graphene-coated all-gold grids after 45 s exposure to residual air glow discharge (negative bias, 15 mA at 0.3 Torr in PELCO easiGlow) (*A*), 45 s exposure to low-power helium plasma (9 W forward power at 1 Torr in Fischione 1070) (*B*), and 4500 s exposure to the same helium plasma (*C*). Scale bar is 10 μm. The grids were covered by a non-contact knife-edge half-mask during the exposure, the approximate location of the mask edge is indicated with the dashed line. The graphene exposed to the glow discharge is destroyed and the non-covered holes in the gold foil appear black (*A*, right). The helium plasma does not affect the structural integrity of the graphene film, even after exposures more than 100 times longer than the typical for functionalization. The shadow of the mask edge is visible across the middle square in (*A*) indicating that the patterning resolution of this masking method is < 50 μm. The insets show 5$\times$ magnified views of the squared areas. The scale bar for the insets is 2 μm.

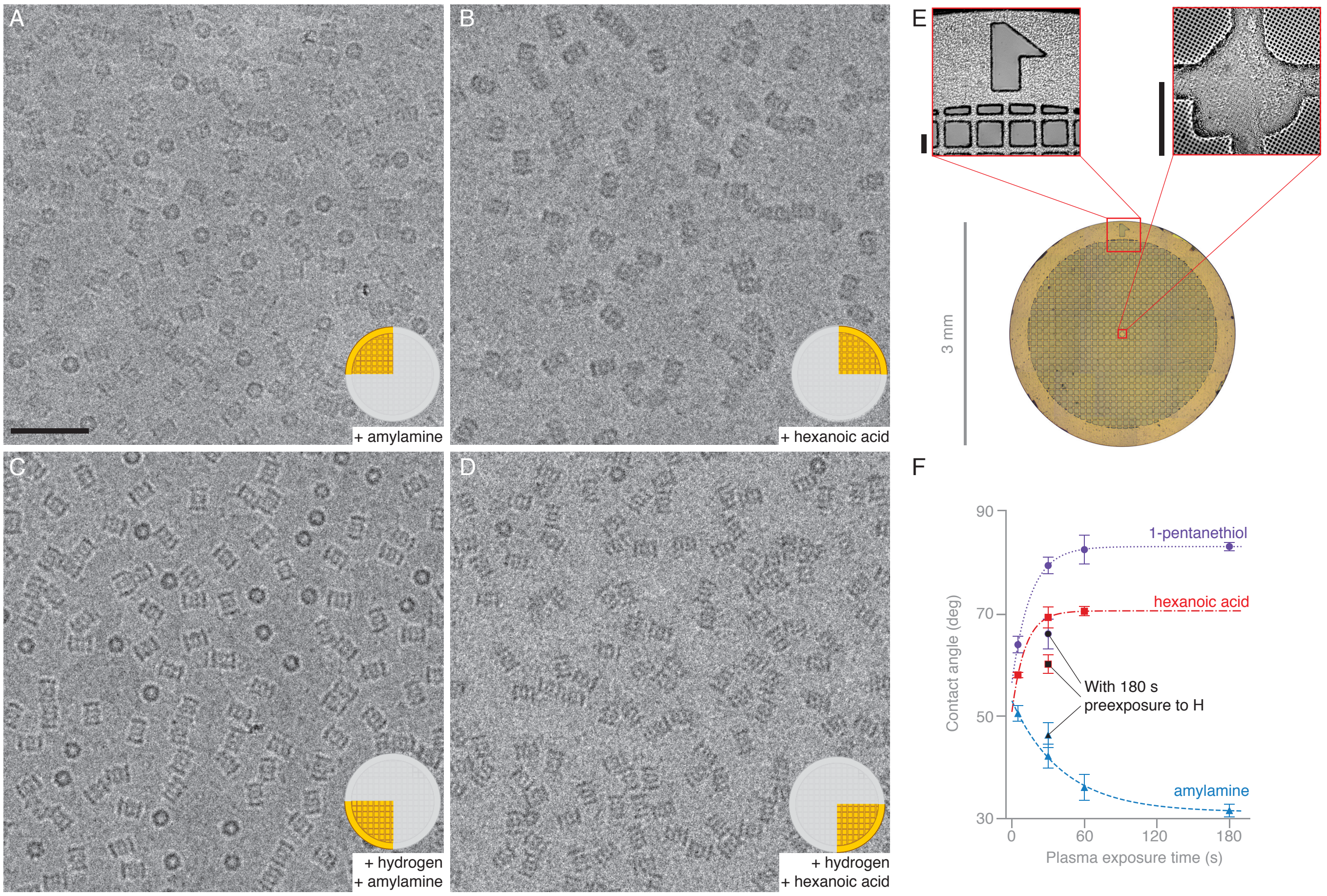

Fig. S6. Controlling the wettability of functionalized graphene and the orientation distribution of 20S proteasomes. Panels (*A* - *D*) show representative micrographs of 20S proteasomes on graphene in ice taken from the four quadrants of a single patterned multifunctional graphene grid. The quadrant of the grid from which the micrograph was collected is highlighted in each inset. The hexanoic acid treatment (*B*) renders the grid hydrophobic which is manifested in the poor wetting of the graphene surface and the protein being partially dried out. This can be rescued by pre-treating the grid with hydrogen plasma (*D*) to make the surface hydrophilic and allow the formation of a uniform thin ice layer independent of the subsequent functionalization. The axes dividing the grid into four differently functionalized quadrants were defined by orienting the grid rim mark (*E*, inset), which can be easily seen by eye, parallel or perpendicular to the mask edge. The quadrants were identified by finding the location and the orientation of the asymmetric grid centre mark (*E*, inset) in low-magnification mode in the TEM. The insets in (*E*) show optical micrographs of the rim mark and the centre mark in their true relative orientation. Scale bars are 500 Å for panels (*A* - *D*), 50 μm for the insets in (*E*). (*F*) Contact angle measurements on functionalized graphene can be used to monitor the extent of surface modification and to assess the hydrophilicity of the resulting surface. Based on previous experiments with partial hydrogenation (2), we estimate that $\sim$ 1 functional group per nm^2 is added to the graphene as a result of a typical exposure. Functionalization with carboxyls and thiols tends to render the surface hydrophobic, as indicated by the higher contact angle, which is problematic for freezing specimens for cryoEM. This can be avoided by a pre-treatment with 180 s of hydrogen plasma before the typical 30 s exposure to the functionalizing chemical (black data points). This method of first partially hydrogenating the graphene before functionalization reliably produced surfaces with sufficient hydrophilicity for preparing cryo specimens.

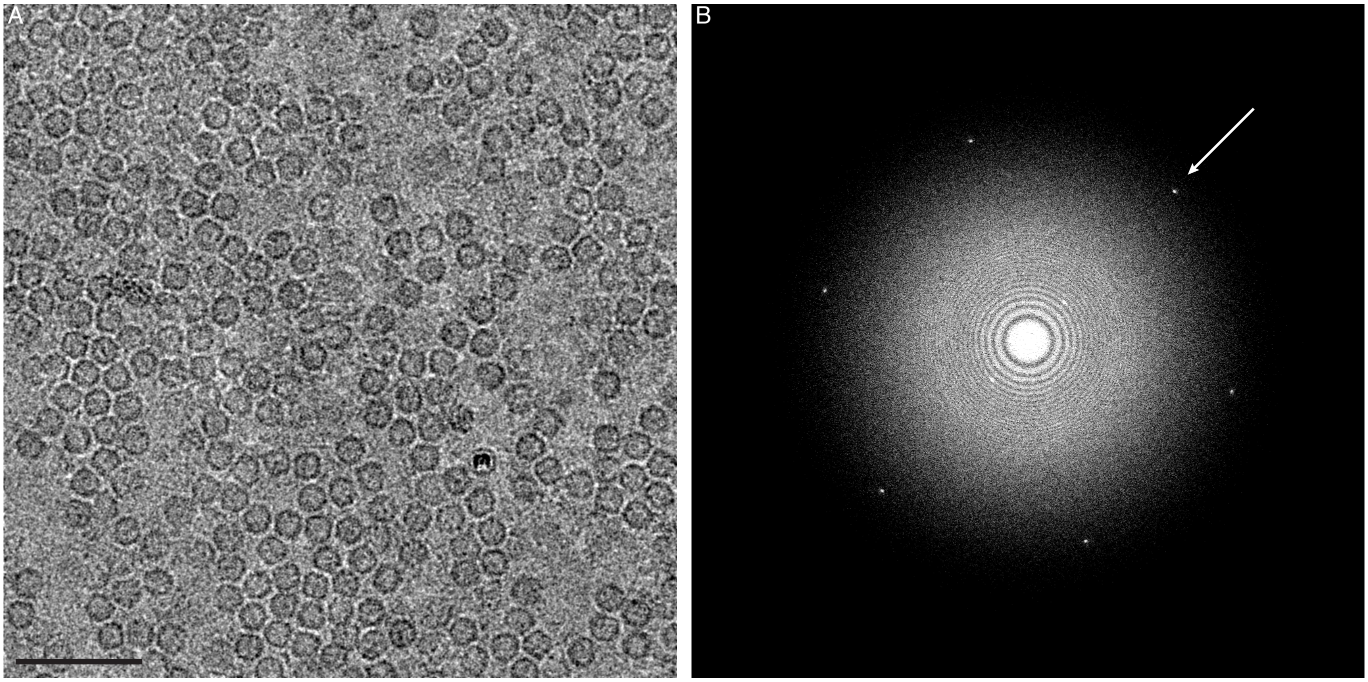

Fig. S7. High-resolution structure determination of apoferritin on graphene-on-gold support. (*A*) Representative micrograph of apoferritin particles in vitreous ice on amylamine-functionalized graphene. Scale bar is 500 Å. (*B*) Fourier transform of the motion-corrected micrograph from panel (*A*). Arrow points to the (110) 2.13 Å graphene lattice reflection, which could potentially be used to further improve motion correction algorithms.

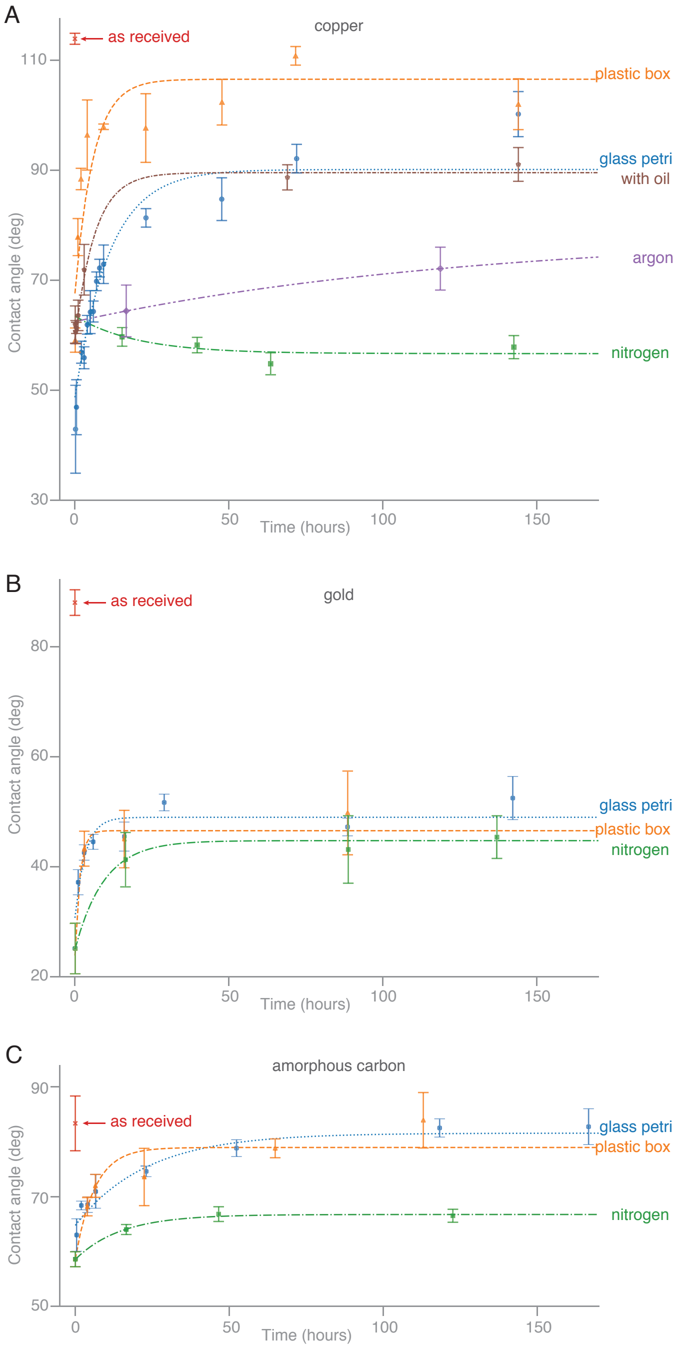

Fig. S8. Storage of plasma-treated specimen supports. The effect of storage conditions on the surface properties of copper (*A*), gold (*B*), and carbon-coated (*C*) grids was measured by means of monitoring their contact angle with water. The contact angle on the as-received grids was measured (*red*), then all grids were exposed to 20 seconds of 9:1 Ar:O_2 plasma and transferred to the respective storage container within $<$ 3 min. The storage conditions tested include: standard plastic grid boxes (*orange*), clean glass petri dishes (*blue*), glass petri dishes contaminated with paraffin oil (*brown*), clean glass petri dishes under continuous flow of clean dry nitrogen gas (*green*), clean containers sealed under argon (*purple*). The change of the contact angle of the grid surface with deionised water was monitored with time after the treatment. All data points are average of n=3 replicates; error bars show standard error in the mean. The lines correspond to exponential fits to the experimental data.

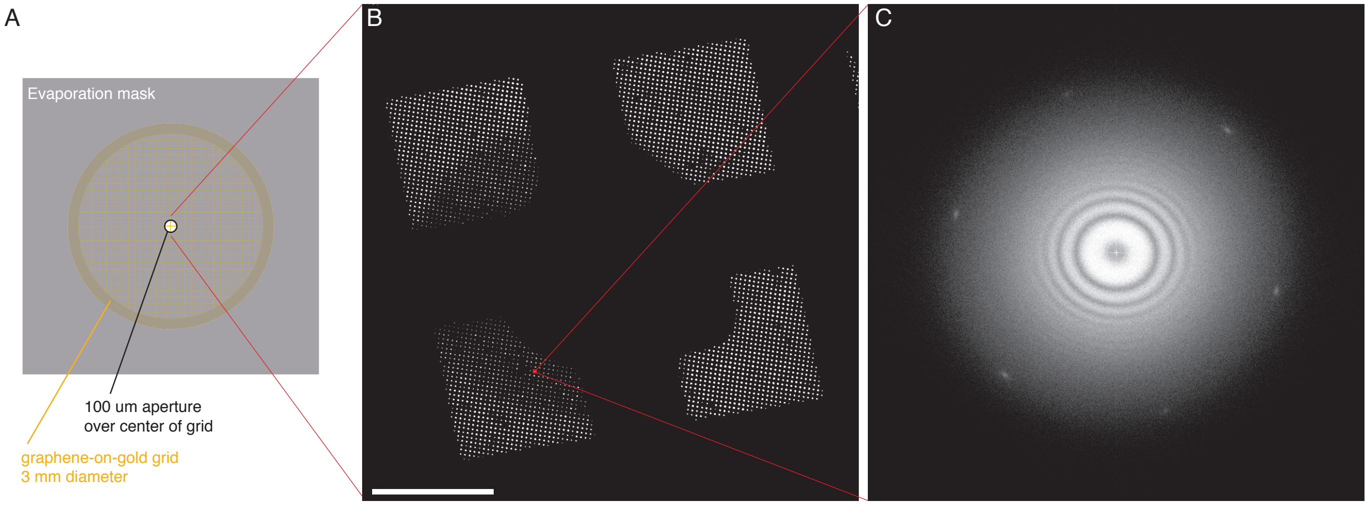

Fig. S9. Multifunctional graphene-on-gold support with an amorphous carbon-covered grid square for electron microscope alignments. (*A*) Diagram of the aperture mask used during carbon evaporation to cover only $\sim$1 grid square of the graphene-on-gold grid with a thin amorphous carbon film. The evaporation is done prior to any plasma treatments. (*B*) Low-magnification transmission electron micrograph of the modified multifunctional grid, showing the asymmetric center mark and the darker region around it that was covered with $\approx$300 Å thick amorphous carbon film. Scale bar is 80 μm. (*C*) Thon rings from the carbon-coated region (Fourier transform of transmission electron micrograph at 155,000$\times$ nominal magnification, 0.4 μm defocus, with 700 Å intentional stigmation) can be used to correct astigmatism and coma. The graphene lattice reflections at 2.13 Å are still visible. Clean monolayer graphene in the areas protected by the mask, in contrast, does not give Thon rings.

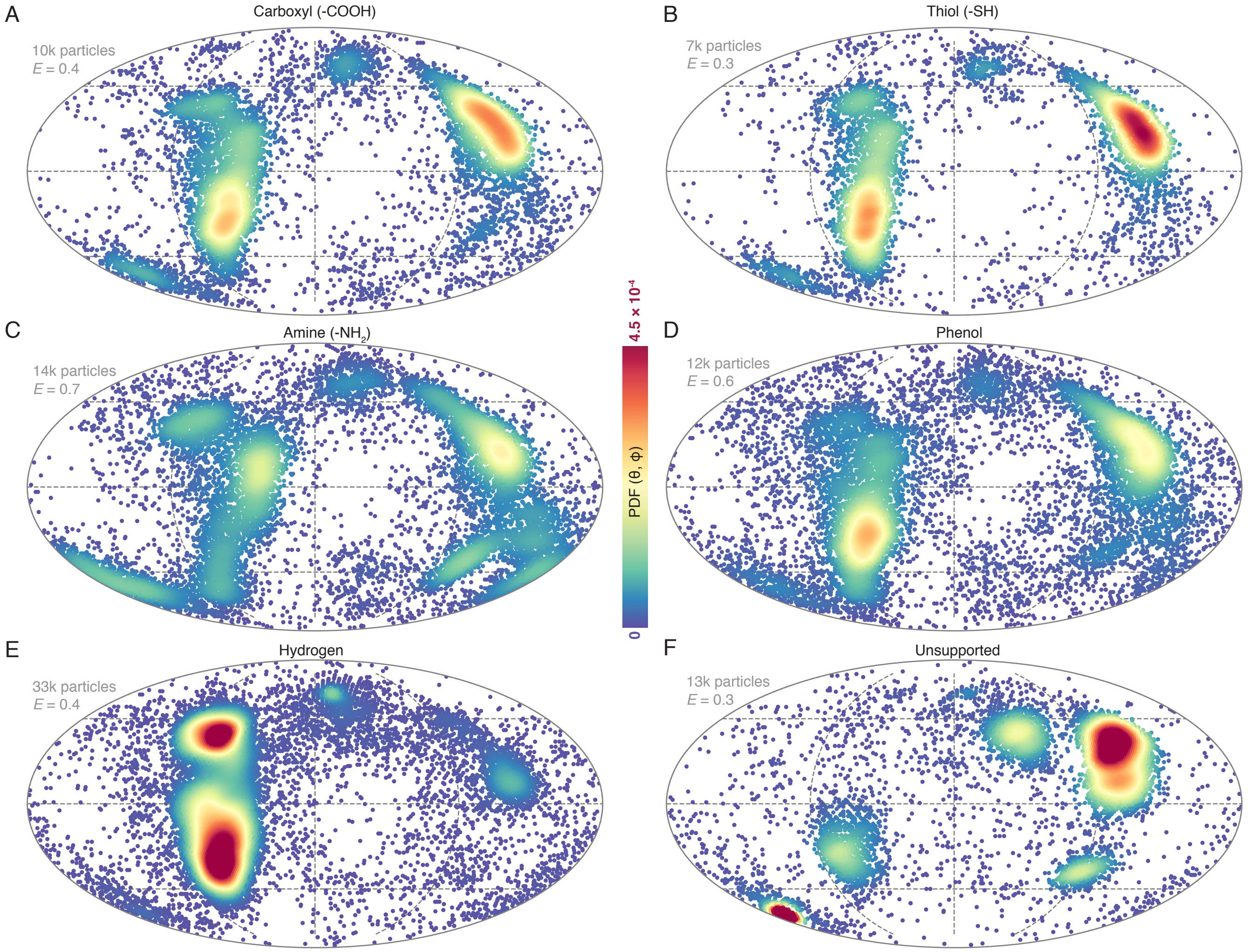

Fig. S10. Controlling the 30S ribosomal subunit orientation distribution on functionalized graphene. Mollweide projection orientation distribution plots of 30S ribosomes on graphene functionalized with hexanoic acid (*A*), 1-pentanethiol (*B*), amylamine (*C*), 4-pentylphenol (*D*), all carried by a helium plasma and preceded by partial hydrogenation, on partially hydrogenated graphene (*E*), and unsupported in ice (*F*). Each point represents an observed particle orientation on the graphene surface and the color scale represents the calculated normalized probability distribution function (PDF) of observing a particle in a view given by the Euler angles (ϕ, θ). All plots are displayed on the same color scale with the peaks corresponding to the dominant views in panels (*E*)-(*F*) saturated. The efficiency E of each orientation distribution is shown.

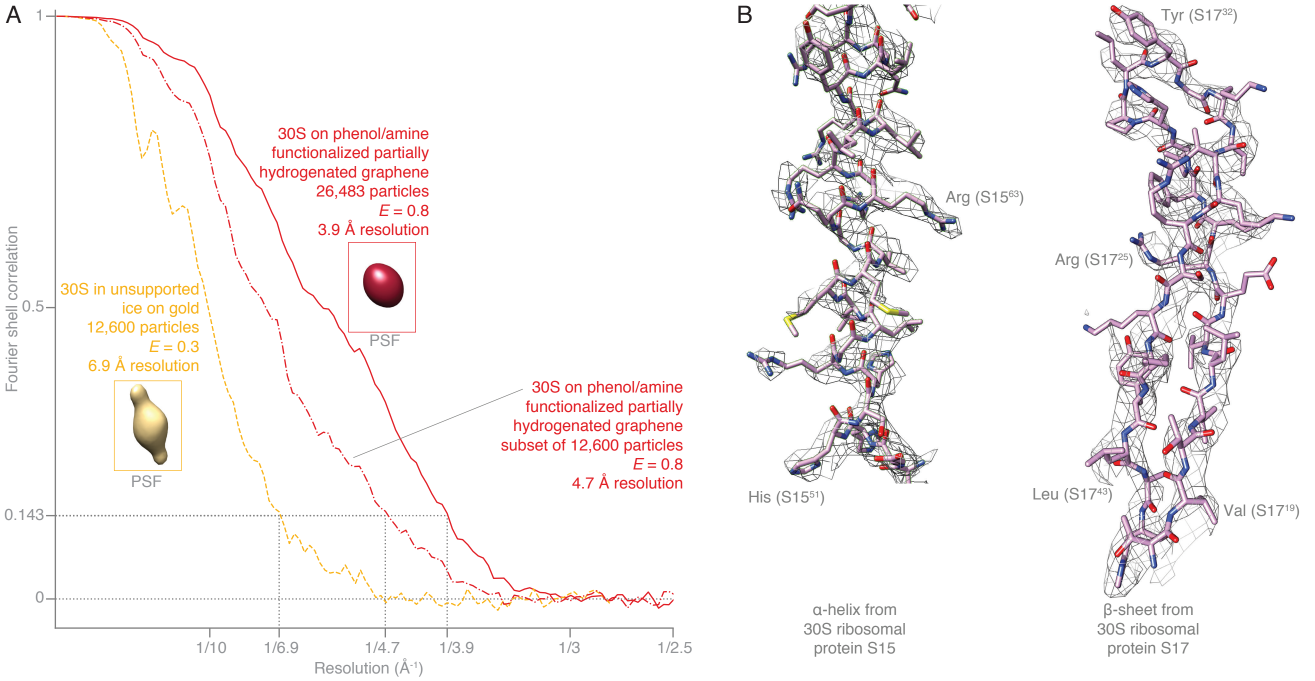

Fig. S11. Efficiency and resolution improvement by optimizing the 30S ribosomal subunit orientation distribution on functionalized graphene. (*A*) Gold-standard FSC plots for the reconstruction of the 30S in unsupported ice (yellow dashed line), yielding 6.9 Å resolution from 12,600 particles, and the 30S on amine/phenol functionalized graphene (red solid line), yielding 3.94 Å resolution from 26,483 particles. The combination of these functionalizations was predicted to maximize the efficiency of the orientation distribution. The difference in resolution is not due to the two-fold difference in particle number, as demonstrated by the reconstruction from a subset of 12,600 particles from the high-efficiency dataset (red dash-dotted line), which reached 4.68 Å. The insets show the corresponding point spread function (PSF) for each orientation distribution; the PSF is elongated in the weakest resolved direction. (*B*) Densities (at level 4σ) from the 3.94 Å map, with the crystal structure of the 30S subunit (1J5E, (3)) fitted using a rigid body fit in Chimera, show clear α-helices and β-sheets, as well as most side chains, as expected at this resolution.

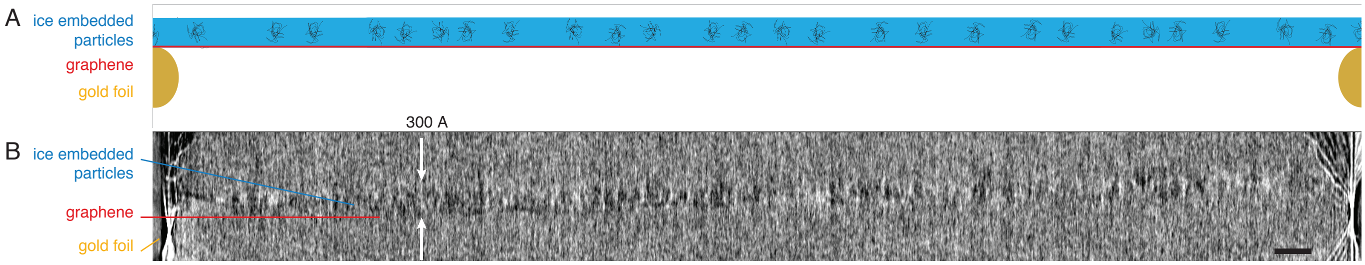

Fig. S12. Tomographic reconstruction confirms that particles within a thin ice layer are adsorbed to the graphene surface. (*A*) Diagram of the specimen on a graphene support. (*B*) Vertical slice through a tomographic reconstruction of a functionalized graphene-covered hole with 30S ribosomes in vitreous ice. The ice thickness is 300 ± 10 Å, just slightly larger than the size of the particles. All particles are located within one layer (see Movie S1 for more detail).

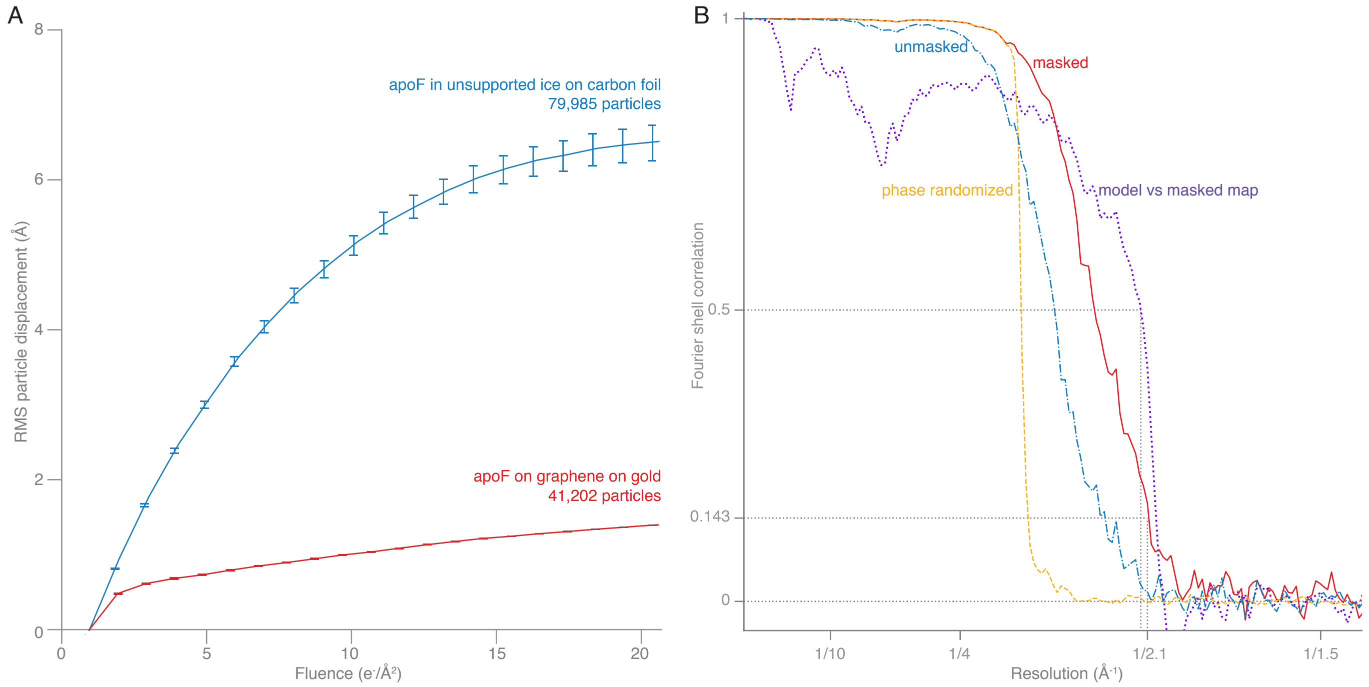

Fig. S13. Facilitated structure determination of apoferritin on graphene-on-gold support due to reduced movement. (*A*) Root-mean-squared (RMS) displacement during irradiation for apoferritin particles in unsupported ice on a carbon grid (blue), and on graphene-on-gold (red). The particles in unsupported ice are from the dataset corresponding to the previously highest-resolution published structure of the commercially available horse spleen apoferritin (4). Error bars are standard error in the mean. The graphene-on-gold support reduces initial movement, the rate of displacement, and the variance in particle motion during irradiation in comparison to holey amorphous carbon supports. (*B*) Fourier shell correlation (FSC) plots for the masked apoferritin half-maps (solid red line), the unmasked half-maps (dash-dotted blue line), the phase randomized half-maps (dashed yellow line), and for the atomic model versus the final masked map (dotted purple line). The final map has an overall resolution of 2.14 Å (0.143 FSC) or 2.19 Å (versus refined structure at 0.5 FSC). All FSCs were calculated in Relion.

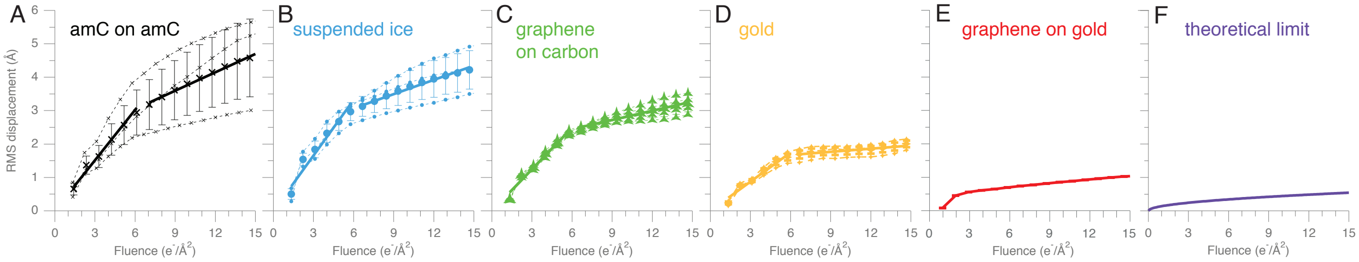

Fig. S14. Reduction of the movement of biological specimens on graphene-coated all-gold grids. Root mean squared (RMS) displacement of ribosomes with MW 2 MDa is plotted versus electron fluence. Panels (*A*) - (*D*) and (*F*) are reproduced from (5). The motion on graphene-on-gold supports (*E*) was originally measured from the apoferritin dataset described in this work, and the effective diffusion constant was scaled by its molecular weight (MW$^{1/3}$) for this comparison, according to the Stokes-Einstein equation (6). The motion of the specimen on a graphene-on-gold support is diffusion-like (with the mean squared displacement directly proportional to the fluence) except at the very beginning of irradiation.

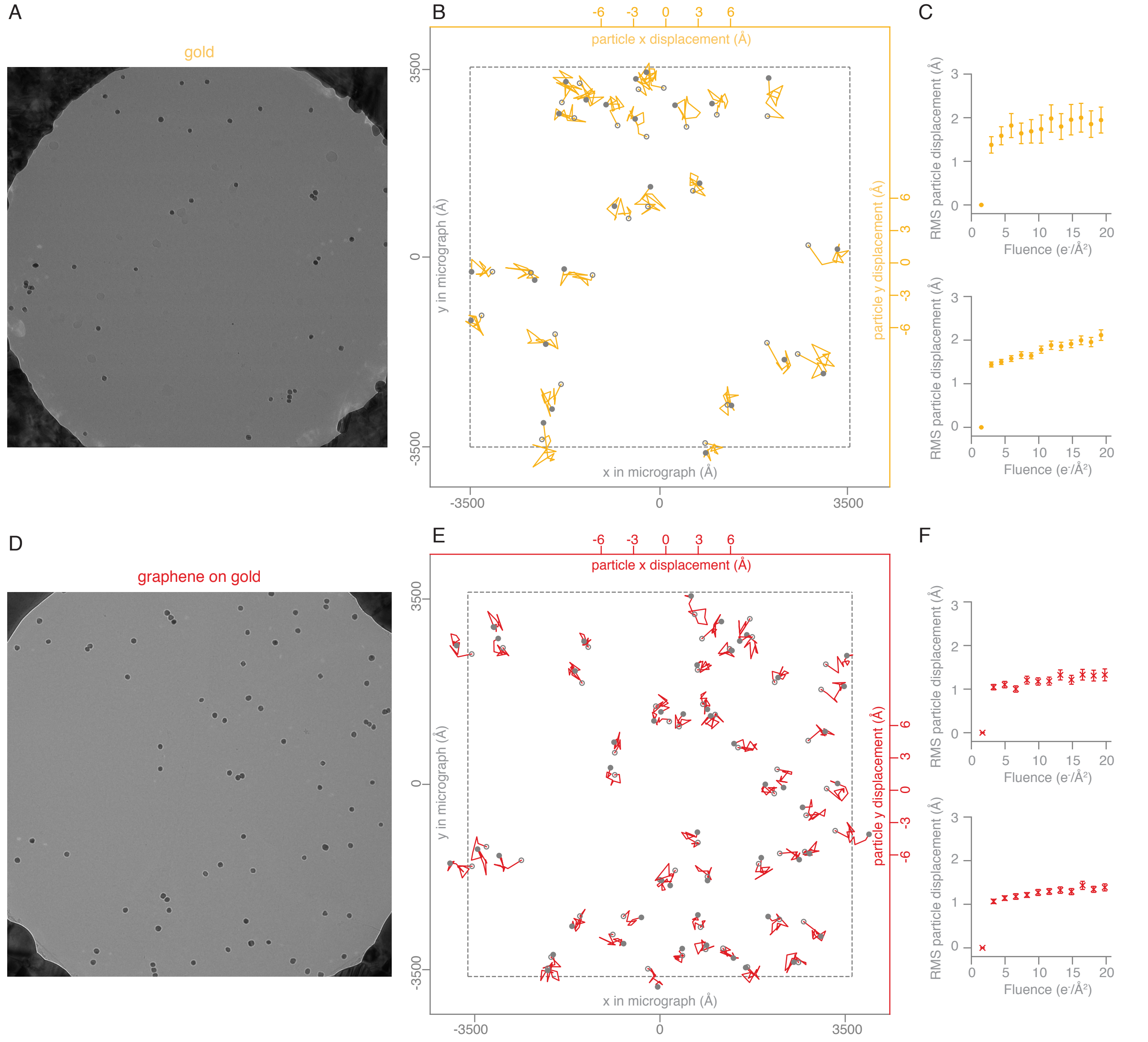

Fig. S15. Reduction of the movement of nanoparticles on graphene-coated all-gold grids. Gold nanoparticles (10 nm in diameter, 6 MDa in mass) were imaged in unsupported vitreous ice on all-gold grids (*A*) and in vitreous ice on graphene on all-gold grids (*D*) at such a magnification so as to include the edges of the hole in the field of view for drift tracking. The trajectories of the particles on the motion-corrected whole micrograph are plotted magnified by 200× (*B*, *E*). The dashed square corresponds to the area of the micrograph, the empty circles are the initial particle positions and the filled circles are the final particle positions after 20 $e^{-}/Å^{2}$ total fluence. (*C*, *F*) The root mean squared (RMS) displacement of the gold nanoparticles is plotted versus electron fluence for the shown micrograph (upper panel) and for an average of $n = 19$ micrographs (gold) and $n = 8$ micrographs (graphene-on-gold) of the same kind (lower panel).

Table S1. Structure and physical properties of compounds tested for graphene functionalization ([7])

Name	Structure	MW (Da)	Vapor pressure at 25°C (Torr)
pentylamine	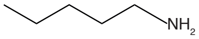	87.16	30
1-pentanethiol	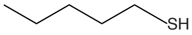	104.21	14
hexanoic acid	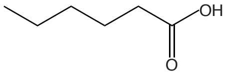	116.16	0.2
4-pentylphenol	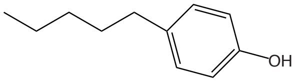	164.25	~ 0.02

Table S2. Data collection statistics for apoferritin

Number of micrographs	479
Number of particles	41 202
Number of asymmetric units	988 848
Pixel size	0.6495 Å
Defocus range	0.4 - 2.0 μm
Mean defocus	1.1 μm
Electron flux	1.23 e^-/Å^2/s
Electron fluence per frame	0.97 e^-/Å^2
Specimen temperature	≈ 80K
Particle box size	(340px)^2
**Model composition**	
Number of asymmetric units (Symmetry)	24 (432)
Non-hydrogen atoms	33000
Protein residues	4104
**Refinement**	
Resolution range	220 - 2 Å
R-factor	0.3484
Fourier shell correlation (map vs. refined model at 0.5)	2.18 Å
**RMS deviations**	
Bonds	0.0325 Å
Angles	4.90 °
**Validation**	
Clashscore, all atoms	13.36
Favored rotamers	82.47%
**Ramachandran plot**	
Favored	96.45%
Outliers	0.59%

Movie S1. Bidirectional tilt series from -50° to $+50^\circ$ in 5° angular increment of 30S ribosomes in ice on functionalized graphene on gold with 800 nm diameter holes. These data were used to calculate the tomogram in SI Appendix, Fig. S12.

References

1. Meyer JC, et al. (2007) The structure of suspended graphene sheets. *Nature* 446:60–63.
2. Russo CJ, Passmore LA (2014) Controlling protein adsorption on graphene for cryo-EM using low-energy hydrogen plasmas. *Nat. Methods* 11(6):649–652.
3. Wimberly B, et al. (2000) Structure of the 30S ribosomal subunit. *Nature* 407:327–339.
4. Zivanov J, et al. (2018) RELION-3: new tools for automated high-resolution cryo-EM structure determination. *eLife* 7:e42166.
5. Russo C, Passmore LA (2016) Progress towards an optimal specimen support for electron cryomicroscopy. *Current Opinion in Structural Biology* 37:81–89.
6. Einstein A (1905) Uber die von der molekularkinetischen theorie der warme geforderte bewegung von in ruhenden flussigkeiten suspendierten teilchen. *Annalen der Physik* 322:549–560.
7. Daubert TE, Danner RP (1989) *Physical and thermodynamic properties of pure chemicals: data compilation.* (Taylor & Francis, Washington DC).
